# Reduction of blood pressure by store‐operated calcium channel blockers

**DOI:** 10.1111/jcmm.12684

**Published:** 2015-10-16

**Authors:** Yan‐Jun Xu, Vijayan Elimban, Naranjan S. Dhalla

**Affiliations:** ^1^ Institute of Cardiovascular SciencesSt. Boniface Hospital Research CentreDepartment of Physiology and PathophysiologyFaculty of Health SciencesUniversity of ManitobaWinnipegMBCanada

**Keywords:** Ca^2+^ antagonists, antihypertensive agents, intracellular Ca^2+^, cell proliferation, hypertension

## Abstract

The voltage‐operated Ca^2+^ channels (VOCC), which allow Ca^2+^ influx from the extracellular space, are inhibited by anti‐hypertensive agents such as verapamil and nifedipine. The Ca^2+^ entering from outside into the cell triggers Ca^2+^ release from the sarcoplasmic reticulum (SR) stores. To refill the depleted Ca^2+^ stores in the SR, another type of Ca^2+^ channels in the cell membrane, known as store‐operated Ca^2+^ channels (SOCC), are activated. These SOCCs are verapamil and nifedipine resistant, but are SKF 96465 (SK) and gadolinium (Gd^3+^) sensitive. Both SK and Gd^3+^ have been shown to reduce [Ca^2+^]_i_ in the smooth muscle, but their effects on blood pressure have not been reported. Our results demonstrated that both SK and Gd^3+^ produced a dose‐dependent reduction in blood pressure in rat. The combination of SK and verapamil produced an additive action in lowering the blood pressure. Furthermore, SK, but not Gd^3+^ suppressed proliferation of vascular smooth muscle cells in the absence or presence of lysophosphatidic acid (LPA). SK decreased the elevation of [Ca^2+^]_i_ induced by LPA, endothelin‐1 (ET‐1) and angiotensin II (Ang II), but did not affect the norepinephrine (NE)‐evoked increase in [Ca^2+^]_i_. On the other hand, Gd^3+^ inhibited the LPA and Ang II induced change in [Ca^2+^]_i_, but had no effect on the ET‐1 and NE induced increase in [Ca^2+^]_i_. The combination of verapamil and SK abolished the LPA‐ or adenosine‐5′‐triphosphate (ATP)‐induced [Ca^2+^]_i_ augmentation. These results suggest that SOCC inhibitors, like VOCC blocker, may serve as promising drugs for the treatment of hypertension.

## Introduction

In view of the effect of different vasoactive agents such as Ang II, NE, ET‐1 and LPA on blood pressure 1, 2, 3, a wide variety of receptor‐blocking drugs are being used for the treatment of hypertension. As the vasoactive hormones and agents are known to promote Ca^2+^ entry into the smooth muscle cells 4, 5, several VOCC antagonists or L‐type Ca^2+^ channel antagonists including verapamil are also known to produce beneficial effect in hypertensive cases^6^. The increase in intracellular Ca^2+^ concentration ([Ca^2+^]_i_) in smooth muscle cells is not only dependent upon the entry of extracellular Ca^2+^ but Ca^2+^ released from the intracellular store has also been demonstrated to play a critical role 7. The release of Ca^2+^ from the intracellular Ca^2+^ stores not only contributes to the increase of [Ca^2+^]_i_ directly, but also causes Ca^2+^ influx through voltage‐independent Ca^2+^ channels, which are called Ca^2+^ release‐activated currents or SOCC 8. Although SOCC blockers such as SKF 96365 (SK) and Gd^3+^ have been shown to reduce [Ca^2+^]_i_ in vascular smooth muscle cells (VSMC) 9, the effects of these agents on blood pressure as well as their actions on LPA‐induced elevation of blood pressure have not been reported previously. Although SK has been shown to reduce the ET‐1‐induced vasoconstriction of rat cerebral arteries 10, the effects of this agent on VSMC with respect to cell proliferation and changes in [Ca^2+^]_i_ because of different agonists have not been studied. In view of the fact that hypertension is associated with an increase in smooth muscle cell proliferation and [Ca^2+^]_i_ 11, this study was undertaken to test the action of SK and Gd^3+^ on blood pressure, cell proliferation and [Ca^2+^]_i_. The specificity of the effect of SK was also examined by using various agents such as Ang II, ET‐1, NE and LPA, which are known to increase the [Ca^2+^]_i_ and blood pressure. Some experiments were carried out to investigate the effect of SOCC blocker, SK in combination with VOCC antagonist, verapamil on blood pressure and [Ca^2+^]_i_.

## Materials and methods

The use of animals and experimental protocol were according to the guidelines of the Canadian Council of Animal Care as approved by the Animal Care Committee of the University of Manitoba. SKF 96365 was purchased from Biomol Research Laboratory (Plymouth Meeting, PA, USA). Gadolinium, L‐α‐LPA and verapamil were obtained from Sigma Chemicals (Oakville, ON, Canada); Fura‐2 acetoxymenthyl/ester (Fura 2‐AM) was purchased from Molecular Probes (Eugene, OR, USA); [^3^H] ‐ thymidine was purchased from Amersham (Oakville, ON. Canada). DMEM and foetal bovine serum (FBS) were obtained from Invitrogen (Burlington, ON, Canada), whereas the A10 VSMC line was from the American Type Culture Collection (Manassas, VA, USA).

### Measurement of blood pressure

Male Sprague Dawley rats weighing 250–300 g were anaesthetized by an intraperitoneal injection of ketamine (90 mg/kg) and xylazine (10 mg/kg) mixture. The right carotid artery was exposed and cannulated with a microtip pressure transducer (model SPR‐249; Millar Instruments, Houston, TX, USA). The catheter was inserted carefully into the lumen of the carotid artery, then catheter was secured with a silk ligature around the artery and blood pressure values were recorded using the computer programme AcqKnowledge for Windows 3.5 (Biopac Systems Inc., Goleta, CA, USA). Arterial systolic and diastolic pressures were measured simultaneously 12.

### Cell number count

The cultured A10 VSMC were treated with 0.25% trypsin‐1 mM ethylenediaminetetraacetic acid (EDTA) for 2 min. and were then collected and centrifuged at 240 × g for 5 min. at room temperature. The supernatant was removed and the cells were suspended in *N*‐2‐hydroxyethylpiperazine‐N′‐2‐ethane sulphonic acid (HEPES) buffer; 0.1 ml of 0.4% Trypan blue was added to 0.5 ml cell suspension and kept for 5 min. at room temperature. About 20 μl of cell suspension was filled in the haemocytometer and the non‐stained cells were counted under microscope.

### Measurement of DNA synthesis

A10 VSMC were cultured in DMEM containing 10% FBS and 0.01 mg/ml gentamicin (Gibco, Burlington, ON, Canada) at 37°C with 95% air and 5% CO_2_. DNA synthesis in A10 VSMC was measured by [^3^H]‐thymidine incorporation into the DNA of the cells 13; cells were cultured in 12‐well plates. Before the experiments, cells were incubated in serum‐free DMEM for 20 hrs. Lysophosphatidic acid was added 10 min. after the addition of different inhibitors and after incubation for 4 hrs, 1 μCi [^3^H]‐thymidine was added. The reaction was terminated after 22 hrs by keeping the cell culture plates on the ice and removing the culture medium. The cells were washed three times with 1 ml HEPES buffer (mM/l: NaCl 145, KCl 4.5, CaCl_2_ 1.0, MgSO_4_·7H_2_O 1.0, HEPES 10, glucose 5, bovine serum albumin 0.1%, KH_2_PO_4_ 1.0, pH 7.4). These cells were then incubated for 1 hr in cold 5% trichloroacetic acid on the ice, washed two more times with 0.5 ml HEPES buffer, and incubated with 0.2 ml NaOH (0.5 N) for 1 hr. The aliquots were transferred to scintillation vial. The radioactivity was counted in a Beckman LS 6500 scintillation counter after the addition of 10 ml Cytoscint‐ES (MP Biomedicals, Santa Ana, CA, USA).

### Measurement of [Ca^2+^]_i_

The cultured A10 VSMC were incubated with 0.25% trypsin‐1 mM EDTA for 2 min. and then the cells were harvested and centrifuged at 240 × g for 5 min. at room temperature. The supernatant was discarded and the cells were incubated with 10 μM Fura 2‐AM in HEPES buffer for 40 min. at 37°C. The cells were then washed twice with HEPES buffer and the cell number was adjusted to 0.3 × 10^6^ cells/ml by adding HEPES buffer. The fluorescence intensity of Fura‐2 was determined by a SLM DMX–1100 dual‐wavelength spectrofluorometer (SLM Instruments, Inc, Urbana, IL, USA); the ratio (R) of fluorescence signal at 340/380 (nM) was calculated automatically. The R_max_ and R_min_ values were determined by the addition of 40 μl Triton X‐100 (10%) and 20 μl EGTA (40 mM) to a cuvette with 2 ml cell suspension respectively. The [Ca^2+^]_i_ was calculated according to the following formula: [Ca^2+^]_i_ = 224 × [(R − R_min_)/(R_max_ − R)] × Sf_2_/Sb_2_, where Sf_2_ and Sb_2_ are the fluorescence proportionality coefficients obtained at 380 nm under R_min_ and R_max_ conditions respectively 14.

### Statistical analysis

The data are expressed as mean ± S.E.M. Statistical analysis was performed with the Microcal Origin Version 6 (Microcal Software Inc., Northampton, MA, USA). The data analysis was carried out by one‐way anova analysis, the comparison of mean values of the two groups was performed by Student's *t*‐test. *P* values less than 0.05 were considered to be significantly different.

## Results

### SOCC blockers on blood pressure

**Figure 1:**
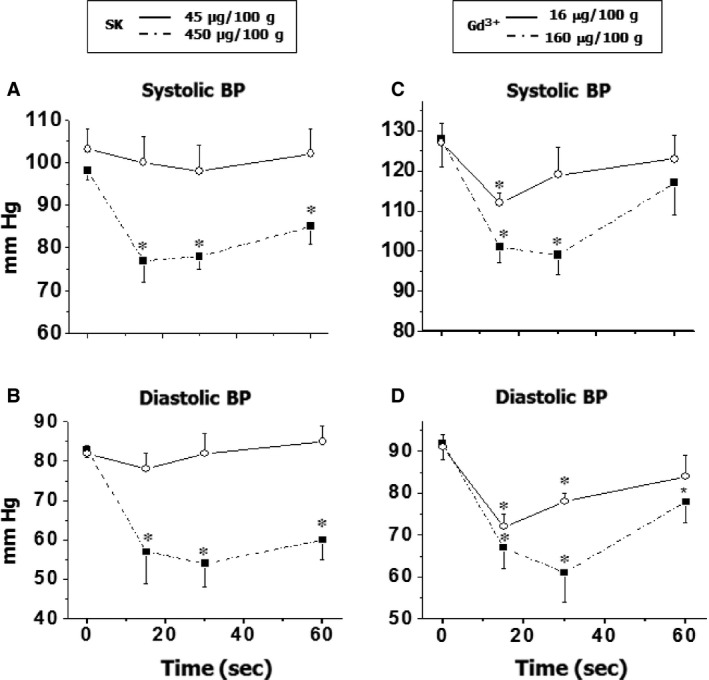
The effect of SK and Gd^3+^ on blood pressure. The blood pressure was recorded from the carotid artery before and after treatment by a microtip pressure transducer and computer programme Acqknowledge for Windows 3.5. *P < 0.05 compared with basal value; *n* = 6.

The effects of SOCC blockers on blood pressure in rats were tested by injecting different doses of SK or Gd^3+^ intravenously. The blood pressure was monitored before and after treatment. In preliminary experiments, both SK (45–450 μg/100 g b.w.) and Gd^3+^ (16–160 μg/100 g b.w.) were found to lower blood pressure in a dose‐dependent manner. As shown in Figure 1, both agents induced dose‐ and time‐dependent reductions in systolic (25% by SK and 23% by Gd^3+^, Figures 1A and 1C, respectively) and diastolic (35% by SK and 33% by Gd^3+^, Figures 1B and 1C, respectively) blood pressures. The maximum effects were achieved within 30 sec. of injection. Both systolic blood pressure and diastolic blood pressure were still significantly lower at 60 sec. of the injection of SK; however, there is no significant difference in the systolic blood pressure at 60 sec. following Gd^3+^ treatment.

**Figure 2:**
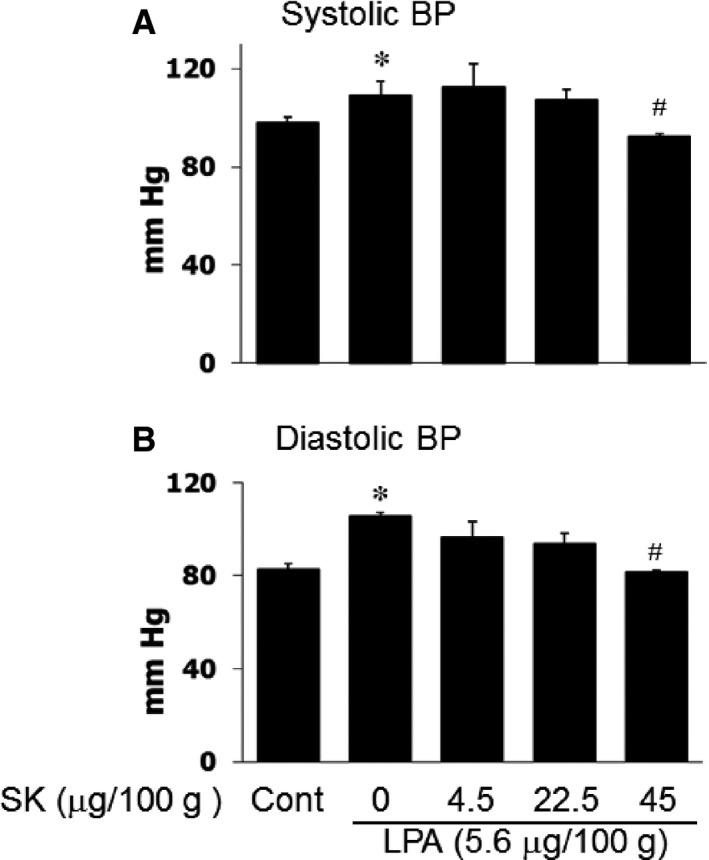
The effect of SK on LPA‐induced blood pressure elevation. The blood pressure was recorded from carotid artery prior to and after treatment by a microtip pressure transducer and computer programme Acqknowledge for Windows 3.5. *P < 0.05 compared to control (Cont) values (basal line), ^#^ *P* < 0.05 compared to LPA injection group but without SK;* n* = 6.

In view of the importance of LPA in the development of hypertension 3, the effect of SOCC blockers on the LPA‐induced elevation of blood pressure were tested by giving different doses of SK (4.5–45 μg/100 g b.w.; i.v.) 30 sec. prior to the treatment with LPA (5.6 μg/100 g b.w.). It was observed that SK pre‐treatment caused a dose‐dependent inhibition of the LPA‐induced change in blood pressure; higher dose of SK (45 μg/100 g b.w.) abolished the effect of LPA (Fig. 2). As VOCC antagonists are commonly used for the control of blood pressure, it was planned to test if the combination use of VOCC and SOCC blockers can produce an additive effect. The blood pressure was monitored before and after injection of SK (45 μg/100 g b.w.), verapamil (15 μg/100 g b.w.) or combination of these two agents. It is pointed out that the use of verapamil in combination with SK was based on the fact that verapamil is well known to block VOCC in both the VSMC and the heart 6, 15 and such a combination could be expected to reduce the side effects of this agent. As shown in Table 1, the combination of SK and verapamil produced a stronger effect on the diastolic blood pressure in comparison to the treatments with SK or verapamil alone.

**Table 1:** Effects of SK (SK&F 96365), verapamil and SK+verapamil combination on blood pressure in rats

Time	SK (*N* = 4)		Verapamil (*N* = 5)		Combination (*N* = 8)	
	SBP	DBP	SBP	DBP	SBP	DBP
0	129 ± 11	85 ± 6	124 ± 7	87 ± 4	134 ± 4	89 ± 2
10 sec.	113 ± 4	74 ± 10	98 ± 3^a^	67 ± 3^a^	96 ± 2^a^	54 ± 2^a^^,^ b
30 sec.	120 ± 9	77 ± 7	97 ± 3^a^	66 ± 4^a^	92 ± 2^a^	47 ± 2^a^^,^ b
1 min.	139 ± 12	90 ± 7	99 ± 2^a^	72 ± 1^a^	99 ± 4^a^	61 ± 4^a^^,^ b
5 min.	142 ± 12	91 ± 13	113 ± 10	78 ± 4	128 ± 6	83 ± 4

a *P* < 0.05 compared with the respective control value (before the treatment).

b *P* < 0.05 compared with respective values of verapamil (15 μg/100 g) group and SK (45 μg/100 g) group.

SBP: systolic blood pressure (mm Hg); DBP: diastolic blood pressure (mm Hg).

### SOCC blockers on cell proliferation

**Figure 3:**
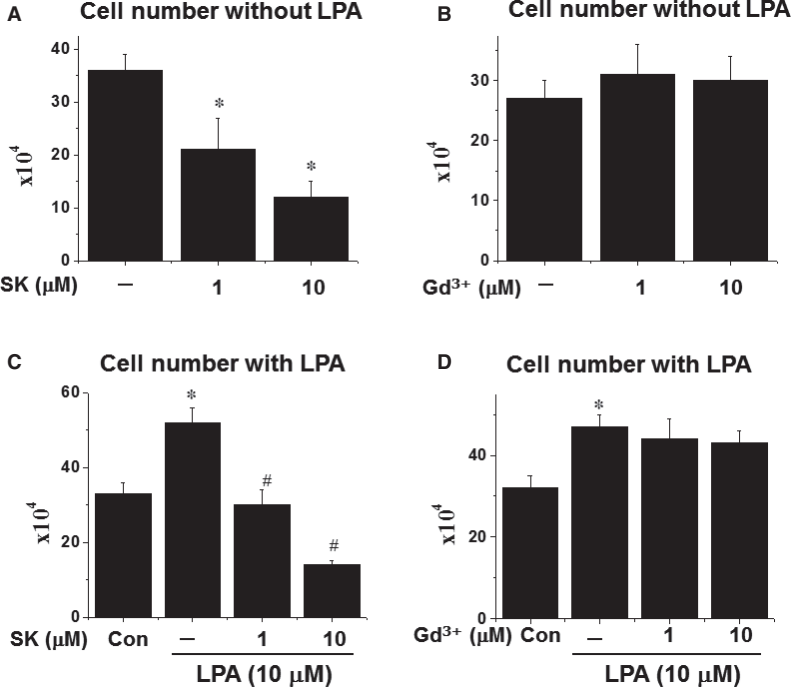
The effect of SK or Gd^3+^ on cell proliferation in the presence or absence of LPA. Prior to treatment, the cells were cultured in serum free medium for 20 hrs, then different concentrations of SK were added to different wells and 10 min. later, LPA was added to all the wells except for control (Con) group. After continuing culture for 24 hrs, the cell number was counted. *P < 0.05 compared to control value; ^#^ *P* < 0.05 compared to the group with LPA but no SK or Gd^3+^; *n* = 6.

**Figure 4:**
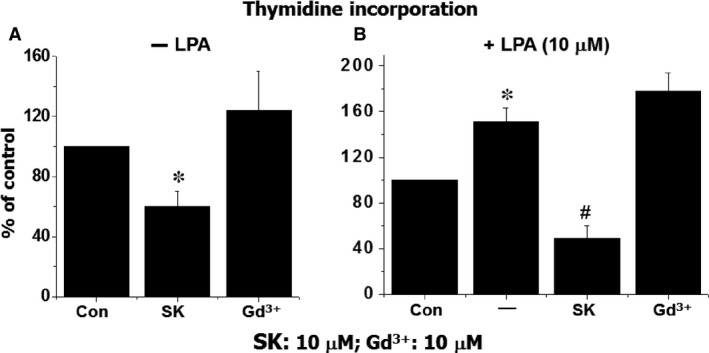
The effect of SK or Gd^3+^ on DNA synthesis in the presence or absence of LPA. Prior to treatment, the cells were cultured in serum free medium for 20 hrs, then different concentration of SK or Gd^3+^ were added to different wells and 10 min. later, LPA was added to all the wells except for control (Con) group. After incubation for 4 hrs, ^3^H‐thymidine was added, then reaction was terminated 20 hrs later. *P < 0.05 compared to control value; ^#^ *P* < 0.05 compared to the group with LPA but no SK or Gd^3+^; *n* = 6.

The ratio of arterial lumen and wall thickness is an essential factor for the regulation of blood pressure 11. As cell proliferation plays critical role in the thickness of blood vessel, the effect of SOCC blockers on cell proliferation was tested in cultured A10 VSMC. As shown in Figure 3, SK caused a dose‐dependent inhibition of cell proliferation in the absence or presence of LPA as reflected by the change in cell numbers; however, Gd^3+^ treatment had no significant effect on cell number. To confirm this finding, [^3^H] thymidine incorporation, an index of DNA synthesis and cell proliferation, was examined in cultured A10 VSMC. As shown in Figure 4, [^3^H] thymidine incorporation with or without LPA was significantly inhibited by SK treatment, meanwhile Gd^3+^ had no significant effect on [^3^H] thymidine incorporation.

### Store‐operated Ca^2+^ channel blocker on intracellular Ca^2+^

**Figure 5:**
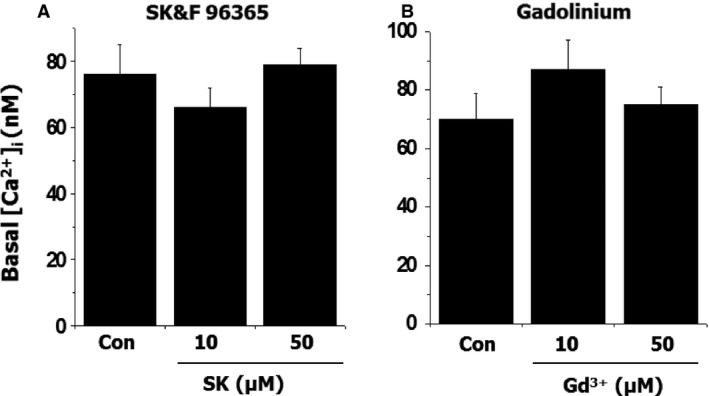
The effect of SK or Gd^3+^ on intracellular free Ca^2+^ concentration in vascular smooth muscle cells. The cells were incubated with 10 μM Fura 2‐AM in HEPES buffer for 30 min. The intensity of fluorescence before and after treatment was recorded by a SLM DMX ‐1100 dual wavelength spectrofluorometer; *n* = 6.

**Figure 6:**
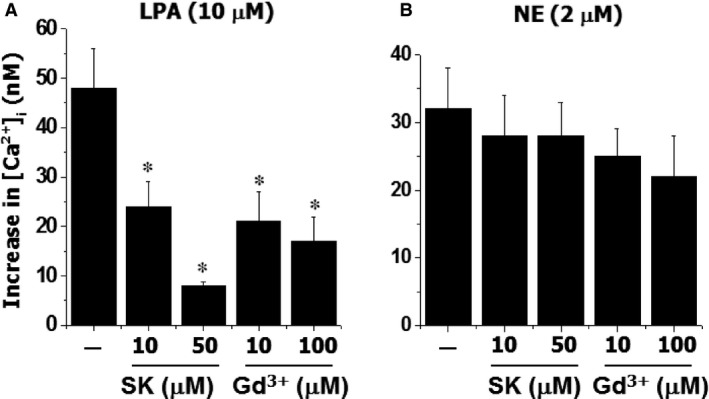
The effect of SK or Gd^3+^ on LPA or NE‐induced change in intracellular free Ca^2+^ concentration in vascular smooth muscle cells. The cells labelled with Fura 2‐AM were incubated with different concentrations of SK or Gd^3+^ for 30 sec. prior to the challenging with LPA or NE. *P < 0.05 compared to control value with LPA or NE alone; *n* = 6.

**Figure 7:**
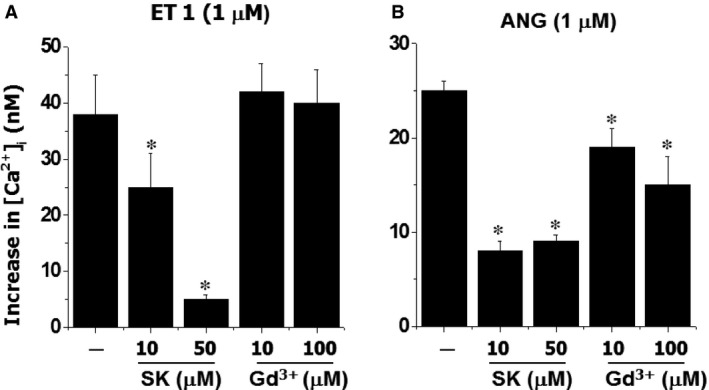
The effect of SK or Gd^3+^ on ET 1 or ANG‐induced change in intracellular free Ca^2+^ concentration in vascular smooth muscle cells. The cells labelled with Fura 2‐AM were incubated with different concentrations of SK or Gd^3+^ for 30 sec. prior to the challenging with ET 1 or ANG. *P < 0.05 compared to control value with ET 1 or ANG alone; *n* = 6.

Intracellular Ca^2+^ concentration is a key factor for the control of vascular tone as well as cell proliferation 16. To examine the effects of SOCC blockers on intracellular calcium mobilization, the effects of SK and Gd^3+^ on the various vasoactive agonists (such as LPA, ATP, NE, Ang II and ET‐1)‐evoked changes in [Ca^2+^]_i_ were examined in cultured A10 VSMC. Figure 5 demonstrated that SK and Gd^3+^ had no significant effect on basal [Ca^2+^]_i_; however, LPA‐induced increase in [Ca^2+^]_i_ was inhibited by both SK and Gd^3+^ in a concentration‐dependent manner. But these antagonists had no significant effect on NE‐induced increase in [Ca^2+^]_i_ (Fig. 6). For ET‐1‐induced elevation of [Ca^2+^]_i_, SK demonstrated a concentration‐dependent suppressive effect; whereas, Gd^3+^ had no significant action (Fig. 7). On the other hand, for Ang II‐induced increase in [Ca^2+^]_i_, both SK and Gd^3+^ exerted inhibitory effects.

### Combination of store‐operated Ca^2+^ channel blocker and voltage‐dependent Ca^2+^ channel blocker on [Ca^2+^]_i_

**Figure 8:**
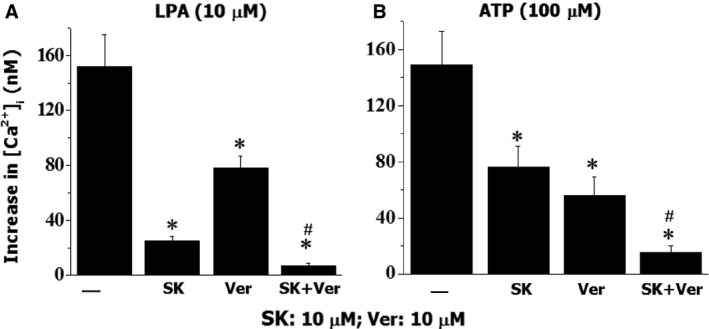
The effect of SK, verapamil (Ver) or SK + Ver on LPA‐ or ATP‐induced changes in intracellular free Ca^2+^ concentration in vascular smooth muscle cells. The cells labelled with Fura 2‐AM were incubated with SK, Ver or SK+Ver for 30 sec. prior to the challenging with LPA or ATP. *P < 0.05 compared to control value with LPA or ATP alone without inhibitors, ^#^ *P* < 0.05 compared to other groups; *n* = 6.

The mobilization of intracellular Ca^2+^ is elicited by Ca^2+^ influx through VOCC, SOCC, receptor operated Ca^2+^ channel (ROCC) and Ca^2+^ release from intracellular store 17. To examine if the combination of SOCC and VOCC blockers is more effective than the individual agent, the actions of SK and verapamil alone or in combination were examined on the LPA‐ or ATP‐induced increase in [Ca^2+^]_i_ (Fig. 8). Both SK and verapamil suppressed the LPA‐ or ATP‐induced elevation of [Ca^2+^]_i_ in A10 VSMC significantly; the combination of SK and verapamil demonstrated a stronger inhibitory effect; the LPA‐induced response was inhibited by 85%, 51% and 95% following the treatment with SK, verapamil and combination of SK and verapamil respectively. For the ATP‐induced response, the inhibitory actions were: 49% by SK, 62% by verapamil and 91% by SK plus verapamil. The reason for choosing LPA and ATP as agonists in this experiment is based on the fact that these agents are broadly used for Ca^2+^ mobilization in VSMC 14.

## Discussion

Hypertension is a major cause of many cardiovascular diseases such as stroke, heart attack, atherosclerosis, chronic heart failure and kidney failure. These cardiovascular diseases affect more than 65 million Americans 18. Voltage‐operated Ca^2+^ channels blockers, including verapamil, nifedipine and diltiazem are useful agents for the reduction of blood pressure in the majority of population; however, some patients do not respond to these drugs. In addition, the major side effect of VOCC blockers is the increase of heart failure incidence as the heart beat is dependent on the Ca^2+^ influx from extracellular space through VOCC. Blocking these channels would reduce cardiac contractility and eventually lead to heart failure 15 and this effect may limit their clinical use in hypertension. On the other hand, Ca^2+^ influx through SOCC is triggered by hormones, growth factors and neurotransmitters. The route of Ca^2+^ influx through SOCC provides a minimal contribution to the beat‐to‐beat Ca^2+^ transients in the heart, but plays a major role in the increase of [Ca^2+^]_i_ in VSMC 19, 20. Thus, SOCC blockers may have less side effects on cardiac contractility when used for the treatment of hypertensive patients.

It is known that Ang II, ET‐1 and LPA are involved in the development of hypertension and atherosclerosis 3. Voltage‐operated Ca^2+^ channels blockers have only a partial inhibitory effect on ET‐1‐induced increase in [Ca^2+^]_i_ and cell contraction 21. Arun *et al*. 22 have reported that verapamil and diltiazem do not inhibit the Ang II‐induced contraction of vascular smooth muscle, indicating that L‐type Ca^2+^ channels are not involved in this response. In an experiment using rat tail artery, the Ang II‐ and ET‐1‐induced contraction was found to be insensitive to L‐type Ca^2+^ channel antagonist, nifedipine, but blocked by SK, indicating that SK may be useful for the treatment of hypertension in patients insensitive to L‐type calcium channel blocker 23. Bova *et al*. 24 demonstrated that norbormide, a vasoconstrictor agent for rat small artery, caused a concentration‐dependent contraction, which was not sensitive to verapamil, but was almost completely inhibited by 30 μM SK. These reports suggest that SOCC blockers may be superior to VOCC antagonists in certain cases.

In this study, we have observed for the first time that SOCC blocker SK reduced blood pressure significantly, and SK suppressed the LPA‐induced elevation of blood pressure. We have also shown the reduction of blood pressure by Gd^3+^, another SOCC blocker 9. These results indicate that SOCC blockers may be used for the control of blood pressure. It should be noted that because the ET‐1‐induced pulmonary contraction was partially blocked by SK and Gd^3+^, different investigators 25, 26 have reported the role of SOCCs in the pathogenesis of pulmonary hypertension. However, some caution should be exercised about this viewpoint regarding the use of SOCC blockers in the treatment of pulmonary hypertension because Gd^3+^, unlike SK, was unable to block the ET‐1‐induced increase in [Ca^2+^]_i_ in VSMC. It is also pointed out that SK and Gd^3+^ produced toxic effects in the body 27, thus SOCC blockers with low toxic and less side effects need to be developed. Zuo *et al*. 28 have demonstrated that tyrosine kinase inhibitors depressed the SOCC activity and it is possible to use tyrosine kinase inhibitors to reduce blood pressure. Accordingly, it would be interesting to investigate the effects of SK and Gd^3+^ on the tyrosine kinase activity in VSMC.

It has been shown that SOCCs play an important role in mitogenic response to growth factor in VSMC 20, and the effect of L‐type Ca^2+^ channel blocker in this regard are controversial. Xiao *et al*. 29 reported that ET‐1 caused a concentration‐dependent increase in cell count and [^3^H] thymidine incorporation in VSMC; both nifedipine and SK inhibited these responses. However, Kawanabe *et al*. 30 reported that nifedipine had no effect on ET‐1‐induced augmentation of [Ca^2+^]_i_ in sustained phase and ET‐1‐stimulated cell proliferation. As SK significantly inhibited both changes in [Ca^2+^]_i_ and cell growth, it appears that SOCC inhibitors may prevent remodelling of VSMC. This view is consistent with the observations of Leung *et al*. 20, who have shown the role of SOCC in VSMC proliferation. Although both SK and Gd^3+^ produce a reduction in blood pressure, these agents were found to exert different actions with respect to VSMC proliferation and [Ca^2+^]_i_ changes. In this regard, it should be noted that there are three important components in SOCC, namely, a stromal interaction molecule (which is Ca^2+^ sensor), a pore forming protein (which allows Ca^2+^ influx) and a transient receptor (which regulates the function of SOCC) 31, 32, 33. As all three components of SOCC are important in the control of Ca^2+^ influx in VSMC 32, 33, it is likely that the difference in the actions of SK and Gd^3+^ on cell proliferation and [Ca^2+^]_i_ observed in this study may be as a result of their effects on different targeting sites in SOCC. Because the expression of the transient receptor component of SOCC is up‐regulated in the vasculature of hypertensive rats 33, future studies should be carried out to investigate the effects of different SOCC blockers on this component.

It is noteworthy that both SK and Gd^3+^ have been shown to block Ca^2+^ influx through SOCC in VSMC and their inhibitory effects are agonist and cell‐type dependent. For instance, in arteriolar smooth muscle cells 34, 35, Gd^3+^ produced a concentration‐dependent inhibition of SOCC‐mediated Ca^2+^ entry; however, the effects of SK on cyclopiazonic acid and thapsigargin depletion‐induced SOCC activation were different. In our study, both SK and Gd^3+^ inhibited LPA‐induced alteration of [Ca^2+^]_i_ as observed previously 7; however, it had no significant effect on the increase in [Ca^2+^]_i_, induced by NE. This is because NE evoked the increase in [Ca^2+^]_i_ mainly by cell membrane depolarization 36 and thus VOCC were activated 37. On the other hand, ET‐1 has been shown to increase [Ca^2+^]_i_ through SOCC which was blocked by SK 38; the results in this study are consistent with this observation. Furthermore, Gd^3+^ produced no effect on ET‐1‐induced [Ca^2+^]_i_ change in our experiments, indicating the distinct effect of these two blockers on SOCC in VSMC. Taken together, LPA, Ang II, NE and ET‐1, were all observed to increase in [Ca^2+^]_i_, but the mechanisms seemed to be different.

We have shown that both SK and verapamil, when used in combination, produced a synergistic effect on LPA‐ and ATP‐induced [Ca^2+^]_i_ increase in VSMC. Other investigators have reported an additive inhibitory effect on ET‐1‐induced vasoconstriction in cerebral arteries by using SK and nifedipine (VOCC blocker) 10. These observations suggest that the use of SOCC blockers in combination with VOCC inhibitor may be more effective in lowering the blood pressure. However, extensive studies by using other agonists including ET‐1, Ang II and NE are required for establishing the beneficial effects of the combination of SOCC and VOCC blockers. Another limitation of this study is concerned with the specificity of SK and Gd^3+^ on SOCC. In this regard, it has been reported that these two agents in higher concentrations may also inhibit ROCC and other Ca^2+^ channels 39, 40, 41. Thus more specific chemical inhibitors or specific antibodies are needed for further study. Nevertheless, on the basis of the results described in this study, it is suggested that SOCC blockers may be considered as a new category of antihypertensive agents and SOCC may be a molecular target for drug development.

## Conflicts of interest

The authors declare no conflict of interest with any grant funding agency.
